# The Functional Erythropoetin rs1617640 Gene Polymorphism does not Affect Life Expectancy of Patients with Peripheral Arterial Disease

**DOI:** 10.31083/j.rcm2407199

**Published:** 2023-07-13

**Authors:** Wilfried Renner, Uwe Langsenlehner, Tanja Langsenlehner

**Affiliations:** ^1^Clinical Institute of Medical and Chemical Laboratory Diagnostics, Medical University of Graz, 8036 Graz, Austria; ^2^Internal Outpatient Department, Prodoc Plus, 8036 Graz, Austria; ^3^Department of Therapeutic Radiology and Oncology, Medical University of Graz, 8036 Graz, Austria

**Keywords:** erythropoietin, peripheral artery disease, genetics, survival, epidemiology

## Abstract

**Background:**

A common functional variant (c.-1306A>C, rs1617640) in the gene encoding erythropoietin (*EPO*) has been linked to expression of erythropoietin and markers of erythropoiesis. Aim of the current study was the analysis of the role of this polymorphism for long term survival of patients with peripheral arterial disease (PAD).

**Methods:**

*EPO* genotypes as well as biomarkers for erythropoiesis were analyzed in a cohort of 946 patients with PAD. Survival follow-up was performed 20 years af-ter recruitment of patients.

**Results:**

Twenty years after recruitment, 752 (79.5%) patients were dead, 103 (10.9%) were still alive, and 91 (9.6%) were lost-to-follow up. In a Cox regression analysis including smoking habit, sex, type-2 diabetes, hypercholesterolemia and arterial hypertension, *EPO* genotypes were not associated with overall survival (Hazard ratio 0.63; 95% confidence interval 0.88–1.08, *p* = 0.63).

**Conclusions:**

The functional *EPO* rs1617640 gene polymorphism, irrespective of its association with markers of erythropoiesis, does not affect survival of PAD patients.

## 1. Introduction

Peripheral artery disease (PAD) is a condition where the flow of blood to the muscles and other tissues in the legs is reduced, typically due to atherosclerosis in the arteries of the lower legs [1, 2].

While major risk factors such as smoking, diabetes, hypertension, and hypercholesterolemia are known to increase the risk for PAD, there is also evidence to suggest that independent genetic factors may contribute to its development. Studies conducted on families have indicated that even after accounting for conventional atherosclerosis risk factors, the heritability of PAD susceptibility is estimated to be around 20% [3, 4, 5].

Erythropoietin, a hormone produced in the kidney, is a key regulator of erythropoiesis and angiogenesis [6, 7, 8]. Angiogenesis is initiated by proliferation and migration of endothelial cells, which can lead to the development of a collateral circulation system, which can function as “endogenous bypass vessels”. The system of collateral blood vessels can be beneficial in mitigating the symptoms and progression of PAD and may result in later onset of symptomatic PAD [9, 10, 11]. Increased serum erythropoietin has been proposed as a useful biomarker and positive predictor for coronary collateral development among patients with chronic coronary artery occlusion [12].

A variant in the promoter region of the gene encoding erythropoietin (*EPO* c.-1306A>C, rs1617640), has been linked to *EPO* gene expression as well as erythropoietin levels [13]. *In vitro* studies have shown that the minor rs1617640 C variant is linked to a 25-fold decrease in luciferase reporter expression compared to the major A variant, and concentration of erythropoietin was 7.5-fold lower in vitreous samples of patients carrying the *EPO* CC genotype compared to those of patients carrying the wildtype AA genotype [14].

In a study by Amanzada and coworkers [14] among chronic hepatitis C patients undergoing antiviral treatment, individuals with the *EPO* rs1617640 CC genotype had a weaker rise of erythropoietin levels and a higher likelihood of needing blood transfusions. Furthermore, a recent genome-wide association study across different ethnic groups found that the *EPO* rs1617640 gene variation was significantly associated with red blood cell (RBC) count [15].

However, these findings are in contrast with a previous study by Fan and coworkers [16] who found that the *EPO* C-variant was linked to elevated erythropoietin levels in a dose-dependent manner. Another study reported a higher frequency of the *EPO* C variant in blood donors with elevated hematocrit levels [17]. Additionally, Kästner and coworkers [18] presented data showing that the C-allele was linked to higher activity of the *EPO* gene promoter. These conflicting studies suggest that the role of the *EPO* rs1617640 gene variation in erythropoietin expression may vary depending on the underlying physiological and pathological conditions.

We have previously observed an association of the *EPO* rs1617640 variant with hematocrit, hemoglobin levels and RBC count in PAD patients [19]. Aim of the present study was to analyze the potential role of this genetic variant in long-term survival of PAD patients.

## 2. Materials and Methods

### 2.1 Human Subjects

The current study included 951 patients with PAD who were recruited at the Division of Angiology at the Department of Internal Medicine, University Hospital Graz, Austria, between 1997 and 2000 [19]. To be eligible, patients had to have an ankle-brachial index of less than 0.9 and/or >50% stenosis of the lower limb artery. All patients routinely underwent a clinical interview, physical examination, ankle and brachial systolic pressure measurement with a Doppler ultrasound probe, as well as vascular examination of the leg arteries by duplex scanning. Six patients with PAD were excluded from the present study because no samples for genotyping were available, leaving 945 patients in the study population.

### 2.2 Clinical Examination and Laboratory Methods

The identification of cardiovascular risk factors and cardiovascular disease was done through a combination of medical records from the University Hospital Graz, medical records provided by general practitioners, and self-reported medical and medication history. Measurement of ankle pressure and calculation of the ankle-brachial index was done according to the method described by Sanchez and Veith [20]. In-person interviews were conducted to obtain information on smoking habits and age at first onset of PAD. Diagnosis of diabetes was based on the criteria established by the World Health Organization [21]. Blood specimens were collected in the morning after an overnight fast. Laboratory measurements of RBC count, hematocrit values and hemoglobin were available for 887 (93.9%) subjects.

DNA was extracted from whole blood using a MagNA Pure LC system (Roche, Vienna, Austria). *EPO* genotypes were analyzed using the 5’-exonuclease (TaqMan) method [22]. To ensure the accuracy of the genotyping process, a subset of 96 samples was analyzed twice, and no discrepancies were detected.

Survival follow-up was analysed using electronic medical records from the Medical University of Graz.

### 2.3 Statistics

Statistical analysis was done with IBM SPSS Statistics release 28 (Chicago, IL, USA). The genotype distribution was tested for Hardy-Weinberg equilibrium using a chi-square test. Categorical variables were compared by chi-square test or Kruskal-Wallis test and summarized as percentages. Continuous variables were analyzed by ANOVA and summarized as means ± standard deviation. For analyses of *EPO* genotypes, dummy codes were assigned assuming an allele dose-effect (wildtype AA genotype = 0, AC genotype = 1, CC genotype = 2) for regression analyses. The treshold for statistical significance was defined as *p* < 0.05.

## 3. Results

An overview of demographic and *EPO* genotype data of the study cohort is presented in Table 1. Determination of *EPO* genotypes was successful in 946 (99.2%) PAD patients and showed no deviation from the Hardy–Weinberg equilibrium. All further analyses were based upon the 946 patients with valid *EPO* genotype.

**Table 1.** **Demographic and *EPO* rs1617640 genotype data of peripheral arterial disease (PAD) patients**.

		PAD patients (n = 946)
Age, years		68.4 ± 10.2
Age at onset of PAD, years		64.8 ± 11.1
Male sex		585 (61.9%)
Type 2 diabetes		455 (48.1%)
Smoker (former or current)		591 (62.5%)
Hypercholesteremia		653 (69.1%)
Arterial hypertension		635 (67.2%)
*EPO* genotype		
	AA	356 (37.7%)
	AC	433 (45.8%)
	CC	156 (16.5%)
*EPO* C allele frequency		0.394

EPO, erythropoietin.

**Fig. 1.**
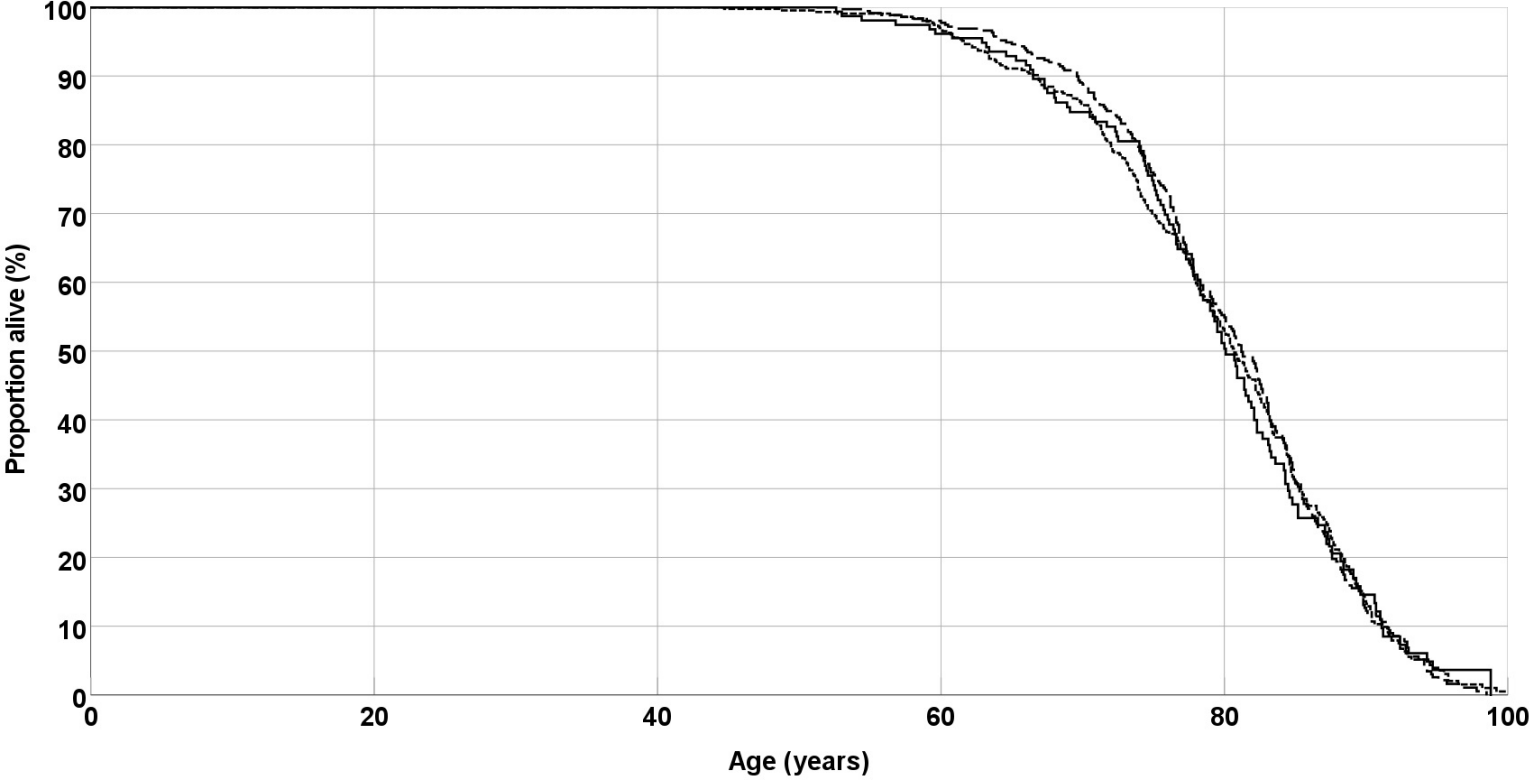
**Overall survival of peripheral arterial disease (PAD) patients**. Lines are separate for *EPO* genotypes AA (solid line), AC (short dashes) and CC (long dashes). *EPO*, erythropoietin.

Twenty years after recruitment of the cohort, 752 (79.5%) patients were dead, 103 (10.9%) were still alive, and 91 (9.6%) were lost-to-follow up. In a Kaplan-Meier analysis, *EPO* genotype was not associated with survival (Fig. 1). Median survival was 80.1 years for the AA genotype, 80.7 years for the AC genotype, and 81.2 years for the CC genotype (*p* = 0.98, Log rank test).

Similarly, in a multivariate Cox regression analysis, which including sex, smoking habit, type-2 diabetes, hypercholesterolemia and arterial hypertension, *EPO* genotypes were not associated with overall survival (Hazard ratio 0.63; 95% confidence interval 0.88–1.08, *p* = 0.63).

## 4. Discussion

We have previously reported that a variant in the promoter region of the *EPO* gene was associated with elevated hematocrit, hemoglobin levels, and RBC count in patients with PAD, as well as age at onset of the disease, making it a candidate genetic risk factor for the disease [19]. In a follow-up analysis 20 years after recruitment of the patients, this *EPO* gene polymorphism showed no association with overall survival.

Erythropoietin is known to be an important regulator of angiogenesis and higher erythropoietin levels in the blood were a positive predictor of collateral formation in patients suffering from coronary artery occlusion [13, 23]. It is possible that the potential favourable angiogenic effects of higher *EPO* expression may have been outweighed by increased erythropoiesis, leading to higher viscosity of the blood and an elevated risk for microvascular complications. It has furthermore to be kept in mind that the observed differences of hematocrit, hemoglobin levels and RBC count between different *EPO* rs1617640 genotype groups were small and did not necessarily indicate severe clinical pathological consequences.

To reduce the chance of false positive results, analyses of *EPO* gene variations was restricted to the rs1617640 polymorphism, which was previously associated with markers of erythropoiesis in PAD patients [19]. Including other non-functional *EPO* variants would inevitably have resulted in a strong decrease of prior probability of association, leading to a higher risk of false positive findings (type I error) [24].

Available medical records included only date of death, but no further information on cause of death. No separate analyses for different causes of death, such as cardiovascular death or cancer-realted death, could be performed.

Furthermore, a part of the initial cohort (n = 91) were lost-to-follow-up with unknown outcome. It is likely that the majority of these patients died before the wide-spread launch of electronic medical records. Due to ethical reasons, survival analysis was restricted to the use of medical records and we were not allowed to approach the patients’ relatives for further survival data.

## 5. Conclusions

In summary, the results of the present study indicate that the functional *EPO* rs1617640 gene polymorphism, irrespective of its association with markers of erythropoiesis and age at onset of the disease, does not affect overall survival of PAD patients.

## Data Availability

The datasets used and/or analyzed during the current study are available from the corresponding author on reasonable request.
